# Randomized Controlled Trials of Proximal Femoral Nail Antirotation in Lateral Decubitus and Supine Position on Treatment of Intertrochanteric Fractures

**DOI:** 10.1155/2013/276015

**Published:** 2013-03-27

**Authors:** Li Xue, Li Zha, Qin Chen, Yi-jian Liang, Kang-ren Li, Zheng Zhou, Jin-long Guan, Hui Qin, You-ping Li

**Affiliations:** ^1^Chinese Evidence-Based Medicine Center, West China Hospital, Sichuan University, Chengdu 610041, China; ^2^Department of Orthopaedics, Chengdu Third People's Hospital, Chengdu 610031, China; ^3^Department of Gynaecology and Obstetrics, Chengdu Women's & Children's Central Hospital, Chengdu 610091, China

## Abstract

The objective of this study was to compare the clinical results and complications of proximal femoral nail antirotation (PFNA) on treatment of intertrochanteric fractures in 120 elderly Chinese patients using Randomized Controlled Trials (RCTs). Totaly 120 cases enrolled were randomly assigned to a lateral decubitus position group and supine position group. The hospital stay, operating time, intraoperative blood loss, length of incision, X-ray fluoroscopy time, and out-of-bed activity time in the lateral decubitus position group were significantly lower than those in the supine position group. There was not statistical significance on union time and Harris values in the two position groups. Moreover, only complications of superficial wound infection were observed in the lateral decubitus position group, but two complications of deep venous thrombosis and wound deep infection were found in the supine position group. The present findings suggested that PFNA applied in elderly patients with intertrochanteric fracture can get satisfactory effects, and the treatment of intertrochanteric fractures using lateral decubitus position showed a satisfactory clinical outcome and a lower radiological complication rate.

## 1. Introduction

Intertrochanteric fractures are defined as “fractures involving upper end of femur through and in between both trochanters with or without extension into upper femoral shaft.” An increasing incidence of intertrochanteric fractures with advancing age is well known, more in men than women, about 1.5 : 1, being outside the joint capsule fracture [1, 2]. The incidence of intertrochanteric fractures varies from country to country. An increase in the elderly population has resulted in a higher incidence of pertrochanteric fractures of the femur. The annual global number of hip fractures is expected to exceed 7 million in the next 40 to 50 years [1, 3]. These fractures will occur more often in the future. Thus, the successful treatment of these fractures is becoming increasingly important part of an orthopaedic practice.

The treatment of these fractures remains a challenge to surgeons. The various treatment options for intertrochanteric fractures are operative and nonoperative. Surgical stabilization with implants is the preferred treatment method for ipsilateral intertrochanteric and femoral shaft fractures, according to most published reports. Various techniques and implants have been developed to manage these complex fractures [1]. Several clinical and biomechanical studies have analyzed the results of different implants such as the dynamic hip screw (DHS), the Gamma nail (GN), and the proximal femoral nail (PFN). Those devices have suffered cutout, implant breakage, femoral shaft fractures and subsequent loss of reduction in the clinical practice [2, 4]. Proximal femoral nail antirotation (PFNA) is a novel intramedullary device with a helical blade that is inserted by impaction, causing bone compaction around the blade; this compaction retards rotation and varus collapse [1, 5, 6]. These characteristics provide optimal anchoring and stability when the implant is inserted into osteoporotic bone. Recent studies have shown favorable clinical results for unstable intertrochanteric fractures treated with PFNA [7–10]. Based on the previous description, PFNA has shared an excellent implant for a wide variety of indications. However, adequate knowledge and experience of operative technique is imperative. Rohilla et al. reported that 41 patients of femoral shaft fractures had closed intramedullary nailing in lateral decubitus position without fracture table or image intensifier, and results suggested that this technique a safe and reliable alternative to achieve closed locked intramedullary nailing without the use of image intensifier and fracture table [11]. Kim et al. have reviewed that patients with femur fractures were treated with closed femoral intramedullary nailing in lateral decubitus position on radiolucent routine table, and results indicated that closed femoral intramedullary nailing in lateral decubitus position with the aid of intraoperative skeletal traction is safe and an effective technique with a low incidence of complications compared to the use of fracture table [12]. Based on the previous results, we hypothesized a lateral decubitus position approach as a simple and convenient technical trick for the treatment of intertrochanteric fractures in elderly patients. To our knowledge, there are few published reports of intertrochanteric fractures treated with PFNA in the lateral decubitus position. The objective of this study was to compare the clinical results and complications of lateral decubitus and supine position PFNA in the treatment of elderly intertrochanteric fracture patients using Randomized Controlled Trials (RCTs).

## 2. Materials and Methods

### 2.1. General Data

A totally of 138 elderly patients with low-energy intertrochanteric fractures were collected and admitted into Department of Orthopaedics, Chengdu Third People's Hospital, from May 2009 to August 2010. Participants were initially screened by telephone, and full assessments were conducted only for participants who did not report any exclusion criteria during the telephone screening. All participants provided written informed consent approved by the Hospital Human Research Ethics Committee.

### 2.2. Diagnosis and Inclusion Standards

In reference to the standard for diagnosing intertrochanteric fractures, the subjects included were (1) those adult men and women of age 60 years old and/or older; (2) those who are confirmed with either by radiographs, computed tomography, or magnetic resonance imaging (MRI); (3) those whose fresh fracture occurred within 21 days; (4) those who are ambulatory prior to fracture; (5) those who signed informed Consent by themselves or their immediate family members; (6) those who have clear consciousness and no severe heart, liver kidney disorders and are able to participate in the examination and treatment. All patients in above lists were excluded.

### 2.3. Exclusion Standards

(1) Those who have old fractures; (2) those who have pathologic fractures and/or caused by osteoporosis; (3) those who have open fractures; (4) those who have multiple injuries or multiple fractures; (5) those who were not suitable for surgery, such as severe decompensated heart, liver, or kidney failure contraindication to the surgery; (6) those who have acute or chronic infection; (7) those who are involved in chemotherapy or hormonotherapy; (8) those who have attended in other trials lately in 6 months; (9) those who are pregnant women; (10) those who are not suitable for the surgery for other reasons by investigators. 

### 2.4. Surgical Procedures

**Figure 1:**
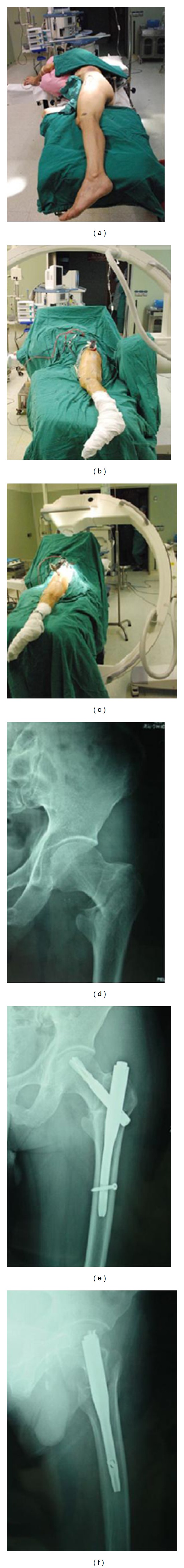
Surgical procedures in the lateral decubitus position. (a) Before treatment, these patients maintained lateral decubitus position, and the disinfection soft pillow was set between the legs, and the healthy limb maintained flexion of their hip and knee. (b) X films are obtained from anterior and posterior positions of hip. (c) C-arm in the “over the top” position to obtain the lateral X films of the hip and maintain approximately 60° with the horizontal plane. (d) Before treatment, X film in intertrochanteric fractures (left side; AO type, 1A1.2); (e) and (f) After treatment, the lateral and anteroposterior X films of the hip are showed, respectively.

Eighteen patients were excluded from 138 elderly patients with low-energy intertrochanteric fractures. One case was excluded for old fracture, two cases were excluded for bonemetastatic tumor, four cases were excluded for multiple fractures, two cases were excluded for chemotherapy or hormonotherapy, and nine cases were not suitable for surgery. The present study is carried out by requirements of double-blind of clinical trials and to achieve separation of collectors of clinical cases, researchers, and statistics evaluators. Thus, the remaining 120 patients were randomized into a lateral decubitus position and supine position group. There was no significant difference statistically in baseline characteristics of two groups. This study was approved by Ethics Committee of Chengdu Third People's Hospital, and all study participants provided both written and verbal informed consent. This study has been registered in the Chinese Clinical Trial Register (no. ChiCTR-TRC-11001815). In the lateral decubitus position group (Figure 1), all surgical operations were carried out under general anesthesia. These patients maintained lateral decubitus position, and injured limb are upper. The disinfection soft pillow was set between the legs, and the healthy limb maintained flexion of their hip and knee during the surgical operations in order to obtain the lateral hip X-ray photos. After anesthesia, the manual traction was operated at the muscle relaxant, and then this operation was stopped. The reset degree was measured by X-ray machine, and the limb was placed in the soft pillow. The proximal trochanteric was slanted to near medial extension curved incision. The abductor muscle fiber was blunt separated, and the guide pin was pointed to the femoral canal midline and introduced in the tip of proximal trochanteric. The pin was measured in the femoral bone marrow cavity, and the hollow reamer was inserted in the proximal. After reamed, the PFNA nail was inserted by designed depth and location requirements under fluoroscopy conditions, and 130-degree aiming arm is connected to the handle. The pin was observed at anterior-posterior position and located in the lower half of the femoral neck; the pin was observed at lateral position and located at the central of femoral neck. The needle tip was located at about 5–10 mm below articular surface. Then, the screw blade was struck along the guide pin by designed location. Drill sleeve was inserted along sight distal locking hole, and the appropriate length locking screw was screwed into the demolition of sigh. Finally, the sealing cap was screwed into the end of the intramedullary nail, and the incision was flushed and closed. In the supine position group (pattern not shown), all surgical operations were also carried out under general anesthesia. These patients maintained supine position, and the torso is slightly slanted to the side slope. The feet were fixed in the boots on the extension device and maintained healthy limb abduction and limb adduction with 10–15° for obtaining lateral X-ray photos. The fractures were reset using continuous mechanical traction and fracture table and maintained this state. The following steps were similar with the lateral decubitus position group.

### 2.5. Evaluation after Treatments

In the present study, all patients received prophylactic antibiotic therapy as follows. 2 grams of cefazolin were given before and after treatments. Also 0.4 mL of low-molecular-weight heparin was administered once everyday up to 14 days. After treatment, the muscles active contraction exercises were carried out by analgesia pump, such as ankle active dorsiflexion and plantar flexion and isometric contraction activity of quadriceps. After one day, joint of lower extremity passive activities was carried out by the assistance of rehabilitation therapists. After 2-3 days, the initiative straight leg and raising, and joint of lower extremity activity were carried out. After 5 days, these patients were allowed to out-of-bed activity. X-ray was measured after 2 weeks, 8 weeks, 12 weeks, 16 weeks, 6 months, 12 months, and 24 months. Patient information, mainly included the duration of hospitalisation, the intraoperative index (operative time, intraoperative blood loss, the length of incision, and the number of intraoperative X-ray), the postoperative index (out-of-bed activity time, the Harris hip score after postoperative 12 weeks, and clinic healing time of fracture), and the surgical complications.

### 2.6. Statistical Analysis

Data were analyzed using Pearson Chi-Square and Wilcoxon-test. For all tests, *P* values <0.05 were considered to be significant. Statistical analysis was performed using the SPSS statistical package, version 15.0 for Windows.

## 3. Results

### 3.1. Baseline Characteristics

The age, sex, injured side, AO type, and complication for patients are shown in Table 1. Out of the 120 elderly patients with low-energy intertrochanteric fractures, 60 patients were fixed with PFNA in the lateral decubitus position and 60 patients in the supine position. In the lateral decubitus position group, the average age was 77.3 years (range 74 to 82). The ratios of male and female patients are 43.3% (26) and 56.7% (34), respectively. The ratios of left and right are 39 : 21. Type A2 fractures were the most common in the study and were seen in 43 patients (71.7%). Type A1 fractures were found in 13 patients (21.7%) and type A3 in 4 (6.6%). The complication of hypertensive disease, diabetes mellitus, and pulmonary disease were 15, 16, and 13 patients, respectively. Similar observation in the supine position group, there was no significant difference statistically in baseline characteristics compared with the supine position group.

### 3.2. Evaluation after Treatments

As shown in Table 2, the mean time of hospital stay for patients treated with PFNA in the lateral decubitus position group was 20.2 d and was significantly lower than in the supine position group. The operative time is significantly different and is 50.6 min in Group A and 65.7 min in Group B. The intraoperative blood loss was 159.2 mL in Group A, while in Group B it was 201.5 mL. The incision length in Group A was 5.9 cm and was significantly shorter than in Group B where it was 8 cm. The number of intraoperative radiation exposure was significantly lower in Group A (8.38) than in Group B (11.5). The median out-of-bed activity time was shorter in Group A than in Group B, being 9.97 days and 12.2 days, respectively. After treatment for 12 weeks, the Harris hip score and average union time of fractures were not statistically different between the two groups.

### 3.3. Complication after Treatments

As shown in the clinical results of complication, three complications were observed in the two groups. The complications were mainly as follows: deep venous thrombosis, superficial wound infection, and wound deep infection (Table 3). One patient developed a superficial wound infection in the lateral decubitus position group, which resolved with intravenous antibiotics. Two complications occurred in the supine position group. One patient developed a deep wound infection and resolved with debridement and intravenous antibiotics treatment. One patient developed deep vein thrombosis and was diagnosed by color Doppler sonography after 1 week postoperatively and treated by the vitamin K antagonist-Warfarin for 3 months.

## 4. Discussion 

In the last few years, the incidence of intertrochanteric fractures has been growing as a result of longer life expectancy owing to better quality of life but also better health care. Many methods have been recommended for the treatment of intertrochanteric fractures [1, 2]. Among them, the PFNA was developed to obtain better fixation strength in the presence of osteoporotic bone, using a simpler technique in comparison with other implants. The inserted PFNA blade achieves an excellent fit through bone compaction and requires less bone removal compared to a screw. Biomechanical tests have demonstrated that the blade has a significantly higher cut-out resistance than commonly used screw systems [13–15]. In the supine group, the traction of limb was continued, and adduction and internal rotation of limb were maintained. These will help in the restoration of fractures and neck shaft angle, as well as the closer fracture chimeric and spin forward foreign greater trochanter. Thus, the trochanteric fossa is revealed, which will help in locating the guide pin. However, the limb and ipsilateral torso remained in a straight line during the actual clinical operation when the patients were supine position. Under the trunk gravitational compression, the convex body lateral soft tissue is particularly evident, especially muscular and/or obese patients. These conditions resulted in adductor and internal rotation of limb and even led anteversion change to besmaller or disappear. The supine position also resulted in the inner and deep shift of intertrochanteric fossa. These changes are often difficult to the positioning guide of main pin during the actual clinical operation. Moreover, if the tail of the guide pin is slight to the outer side, the guide pin will point at inside bone cortex, which will result in false channel formation. Thus, the false channel is needed to correct direction of the guide pin, and the operation time will extend, and intraoperative blood loss and the number of X-ray exposure also increase. Moreover, these patients were placed in the supine position on the flat radiolucent table, which can result in difficult to obtain a good lateral image of the proximal femur [16, 17]. Thus, adequate knowledge and experience of operative technique need further improvement.

Nailing of the femur can be performed in both supine and lateral position. The supine position is physiologic and convenient to the anesthetist and is preferred if patients also have cervical spine injury, ipsilateral lower extremity fracture and severe pulmonary compromise. But access to greater trochanter is somewhat limited in supine position, particularly in large or obese patients in whom lateral position is preferred [16–19]. Thus, lateral positioning obviates the need for a fracture table, makes it easier to establish a starting point for an intramedullary device, and facilitates conversion to an open procedure without repositioning should this become necessary. In the present study, we designed the lateral decubitus position PFNA and investigated and compared the clinical results and complications of PFNA in the treatment of elderly intertrochanteric fracture patients. These patients were placed in the lateral decubitus position on the flat radiolucent table, and the healthy limb maintained flexion of their hip and knee during the surgical operations in order to obtain the lateral hip X-ray photos. Under anesthesia and muscle relaxation conditions, the manual traction was operated in order to restore fractures and will avoid the complications caused by the use of the fracture table, such as pudendal, sciatic and femoral nerve palsy, perineal sloughs, well leg compartment syndromes, avulsion of the inferior epigastric artery in the contralateral limb, and crush syndromes [20]. The present results suggested that only one patient developed a superficial wound infection in the lateral decubitus position group (Table 3). Compared with the supine position group, once surgical operation achieved restore fractures in the lateral decubitus position group. Most of intertrochanteric fractures are low-energy osteoporotic fractures, and primary injuries of soft tissue are lighter [1]. Thus, soft tissue hinge effect may increase antishift capacity between the fracture fragments after restore. Moreover, the lateral decubitus position will help to maintain the neck-shaft angle, restore fracture chimeric, and further improve the nail of guide needle accurately plugged in the medullary cavity. The present findings showed that the lateral decubitus position PFNA can change the overlapping shielding effect of pelvis when the guide pin was inserted, and the location of rotor nest changes shallowly compared to the supine position groups. Manual reduction is only reconsidered as simply axial traction, and it has low-energy injury. Thus, manual reduction can promote and maintain the reduction of soft tissue chain. The advantages of the lateral decubitus position group also mainly included sustained traction, mild adduction, and internal rotation bit of hips, as well as without obvious shift performance of restore fracture. 

After treatments for 12 weeks, the present findings showed the length of hospital stay, the intraoperative parameters (operative time, incision length, intraoperative blood loss, and the number of intra-operative radiation exposure), and out-of-bed activity time were significantly lower (*P* < 0.05) in patients treated with a PFNA in the lateral decubitus position group compared to those treated in the supine position group. However, there was no significant difference in the final functional outcome on treatments of intertrochanteric fractures in the elderly patients. Of course, PFNA with the lateral decubitus position also has some limitations on the treatments of intertrochanteric fractures in the elderly patients, who cannot tolerate the lateral decubitus position. Moreover, some patients have some complications, such as spine unstable fractures, severe lung damage disease, and contralateral lower extremity fractures, who are not suitable for the lateral decubitus position.

## 5. Conclusion

The present results showed that PFNA with the lateral decubitus position and the supine position provided effective methods for the treatments of intertrochanteric fractures in the elderly patients. However, the PFNA with the lateral decubitus position provides a shorter operation time, less of hospital stay, blood loss, number of intraoperative X-ray, incision length and out-of-bed activity time. Therefore, PFNA fixation in the lateral decubitus position may be considered a better choice for the treatment of intertrochanteric fractures in the elderly patients. Although PFNA fixation in the lateral decubitus position was shown to be of value as the treatment of intertrochanteric fractures, further definitive research about early surgery and longer follow-up period is needed to support the use of PFNA fixation in the lateral decubitus position.

## Figures and Tables

**Table 1:** Baseline characteristics in the lateral decubitus position and supine position groups.

No.	Group A (*N* = 60)	Group B (*N* = 60)	Std. deviation	*P* values
Age (years)	77.3 (74~82)	75.7 (71~80)	−1.669*	0.095
Sex (male/female)	26 : 34	29 : 31	0.302**	0.583
Injured side (left/right)	39 : 21	37 : 23	0.144**	0.705
AO type				
31-A1	13	15		
31-A2	43	42	0.297**	0.862
31-A3	4	3		
Complication				
Hypertensive disease	15	12	0.430**	0.512
Diabetes mellitus	16	14	0.178**	0.673
Chronic obstructive pulmonary	13	15	0.186**	0.666

**Pearson Chi-Square, *Wilcoxon-test; Group A: the lateral decubitus position; Group B: the supine position.

**Table 2:** Comparison of scores in the lateral decubitus position and supine position groups after treatments.

No.	Position	Mean	SD	*Z*	*P*
Hospital stay (days)	A	18	2.490	−3.592	0.000
	B	20.2	3.857		
Operation time (min)	A	50.6	12.086	−5.317	0.000
	B	65.67	16.204		
Blood loss (mL)	A	159.2	26.316	−6.235	0.000
	B	201.5	38.038		
Length of incision (cm)	A	5.9	1.362	−6.189	0.000
	B	8	1.727		
X-ray fluoroscopy time	A	8.38	1.949	−6.140	0.000
	B	11.5	2.722		
Out-of-bed activity time (h)	A	9.97	2.365	−4.162	0.000
	B	12.2	2.865		
Harris values	A	83.1	4.466	−1.951	0.051
	B	81.8	4.280		
Union time	A	11.7	2.784	−0.542	0.588
	B	11.9	2.899		

Wilcoxon-test, Group A: the lateral decubitus position; Group B: the supine position.

**Table 3:** Comparison of complication in the lateral decubitus position and supine position groups after treatments.

Complication	Group A (*N* = 59)	Group B (*N* = 56)
Fracture of shaft of femur	0	0
Cutout	0	0
Deep venous thrombosis	0	1
Superficial wound infection	1	0
Wound deep infection	0	1
Nonunion	0	0

Group A: the lateral decubitus position; Group B: the supine position.
